# Antimicrobial Neuropeptides and Their Receptors: Immunoregulator and Therapeutic Targets for Immune Disorders

**DOI:** 10.3390/molecules30030568

**Published:** 2025-01-27

**Authors:** Kaiqi Chen, Xiaojun Wu, Xiaoke Li, Haoxuan Pan, Wenhui Zhang, Jinxi Shang, Yinuo Di, Ruonan Liu, Zhaodi Zheng, Xitan Hou

**Affiliations:** 1 College of Medical Imaging and Laboratory, Jining Medical University, Jining 272067, China; chenmalan@126.com (K.C.); lxk084408@163.com (X.L.); 18764547069@163.com (H.P.); douyuhao0531@163.com (W.Z.); 15762154779@163.com (J.S.); 15854709757@163.com (Y.D.); 2 College of Medical Engineering, Jining Medical University, Jining 272067, China; wuxiaojun9979@163.com (X.W.); 15376731750@163.com (R.L.)

**Keywords:** antimicrobial neuropeptides, immunoregulator, receptors, therapeutic targets, immune disorders

## Abstract

The interaction between the neuroendocrine system and the immune system plays a key role in the onset and progression of various diseases. Neuropeptides, recognized as common biochemical mediators of communication between these systems, are receiving increasing attention because of their potential therapeutic applications in immune-related disorders. Additionally, many neuropeptides share significant similarities with antimicrobial peptides (AMPs), and evidence shows that these antimicrobial neuropeptides are directly involved in innate immunity. This review examines 10 antimicrobial neuropeptides, including pituitary adenylate cyclase-activating polypeptide (PACAP), vasoactive intestinal peptide (VIP), α-melanocyte stimulating hormone (α-MSH), ghrelin, adrenomedullin (AM), neuropeptide Y (NPY), urocortin II (UCN II), calcitonin gene-related peptide (CGRP), substance P (SP), and catestatin (CST). Their expression characteristics and the immunomodulatory mechanisms mediated by their specific receptors are summarized, along with potential drugs targeting these receptors. Future studies should focus on further investigating antimicrobial neuropeptides and advancing the development of related drugs in preclinical and/or clinical studies to improve the treatment of immune-related diseases.

## 1. Introduction

Recent studies have revealed the elaborate crosstalk between the immune and nervous systems [1]. These systems use a shared biochemical language, including neurotransmitters such as neuropeptides and hormones, immune ligands, and their receptors [2]. This interaction forms a bidirectional communication network. Neuropeptides are key players in this process, regulating specific neuroimmune disorders [3].

Antimicrobial peptides (AMPs) serve as the primary line of defense for the host against pathogenic organisms and possess the capacity to elicit innate immune responses [4]. Some neuropeptides produced by neurons, glial cells, and immune cells exhibit physical properties comparable to those of AMPs and have been verified to possess specific antimicrobial properties [5]. Moreover, neuropeptides function as neuroendocrine modulators [6], playing a vital role in the modulation of inflammatory responses [7]. The receptors for these neuropeptides serve as essential targets for immune regulation [7]. Potential therapeutic agents targeting their respective receptors are anticipated to be viable treatments for specific immune disorders and to progress toward clinical therapy [8,9,10].

Neuropeptides that exhibit antimicrobial and immunoregulatory properties have the potential to significantly impact the pathophysiology of various immune-related diseases [11]. This review provides a comprehensive examination of the immune regulatory mechanisms mediated by neuropeptides, along with an analysis of their sources, target cells, and immunoregulatory functions of antimicrobial neuropeptides. Furthermore, we summarize the receptors for the 10 most prominent antimicrobial neuropeptides, including their respective agonists and antagonists, as well as the implications for disease treatment. By leveraging the immune-regulating properties of those antimicrobial neuropeptides, we propose that they may play a substantial therapeutic role in specific diseases.

## 2. Neuropeptide Immunoregulatory Mechanism

**Figure 1:**
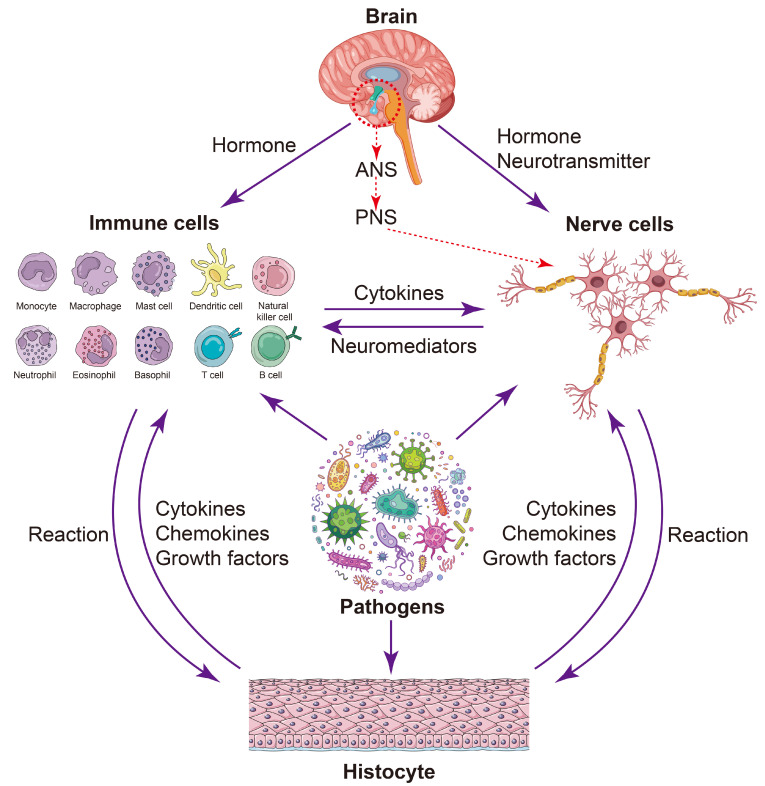
Bidirectional communication between the neuroendocrine and immune system. An intricate interplay exists between the neuroendocrine system and the immune system. Firstly, pathogens can induce immune cells to produce cytokines and nerve cells to release neuromediators, thereby facilitating the exchange of these chemical signals between immune cells and nerve cells. Secondly, pathogens can also stimulate other cell types, such as epithelial cells, leading to the secretion of effector molecules including cytokines, chemokines, and growth factors. These factors can further activate immune cells and nerve cells through receptor-mediated responses to pathogen stimulation. Additionally, the involvement of the hypothalamic–autonomic nervous system axis (HANS) should be considered. For instance, the brain–gut axis has been shown to trigger hypothalamus–pituitary–adrenal function within the central nervous system (CNS). The diagram of the immune cells was refined based on Krause’s work [18]. The diagram of the brain, pathogens, and nerve cells was designed by pch.vector/Freepik.

Traditionally, the neuroendocrine system and the immune system have been viewed as distinct regulatory domains that govern homeostasis between the host and its environment [12]. Each domain is characterized by its own specialized terminology, and only a limited number of researchers possess expertise in both areas [13]. It was not until the late 1970s, with advancements in research into both the immune and neuroendocrine systems, that the complex crosstalk between these two systems was elucidated [12,14]. The neuroendocrine system and the immune system communicate internally through a biological regulatory signal library. Immune cells can respond to stimuli by secreting neurotransmitters, whereas the neuroendocrine system can modulate immune responses through the production of cytokines [13,15,16] (Figure 1). This interplay between the neuroendocrine system and the immune system is essential for pathogen elimination and the re-establishment of immune homeostasis [17]. Neuropeptides, a class of neuroendocrine mediators, are secreted by both immune and nerve cells. The literature has established that neuropeptides act as multifunctional regulators of the immune response [6] and are capable of activating immune cells to elicit either anti-inflammatory or pro-inflammatory effects.

### 2.1. Anti-Inflammatory Effects

The induction of immune tolerance is vital for maintaining immune homeostasis, modulating autoreactive T cells, preventing the onset of autoimmune diseases, and achieving transplant tolerance [7]. Inflammation is a crucial physiological response against pathogen eradication; however, the inadequate regulation of this process can lead to significant adverse effects on the host [7]. Investigating the endogenous factors that influence immune tolerance and inflammation represents a significant research focus within the field of immunology. Between 2000 and 2008, Delgado’s research team reported that neuropeptides synthesized by nerve cells and immune cells have anti-inflammatory properties and facilitate the maintenance of immune homeostasis. These neuropeptides include vasoactive intestinal peptide (VIP), α-melanocyte-stimulating hormone (α-MSH), urocortin I (UCN I), adrenomedullin (AM), and cortistatins [7,12,17].

**Figure 2:**
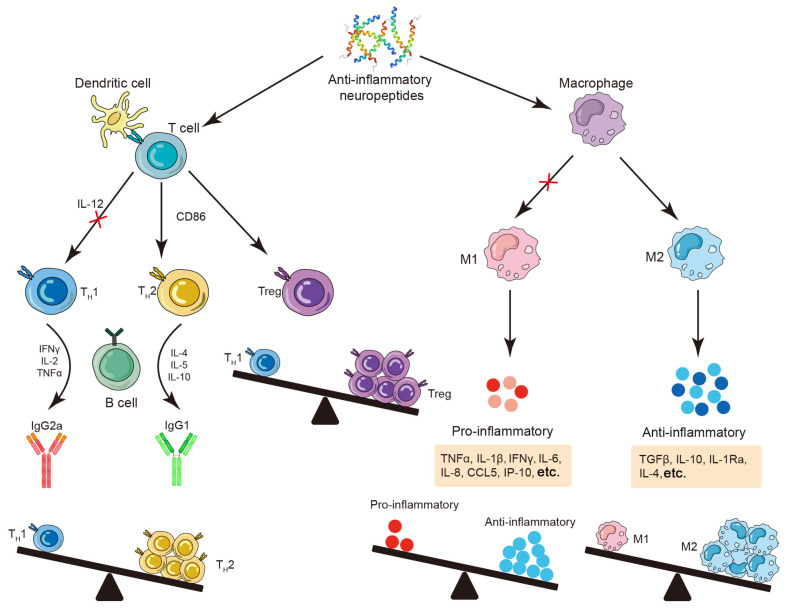
Antimicrobial neuropeptides are key players in anti-inflammation. Certain neuropeptides synthesized by both neuronal and immune cells effectively respond to pathogen stimuli and mediate anti-inflammatory effects. Firstly, neuropeptides can modulate T cell function by inhibiting the production of TH1-related factor IL-12, inducing CD86 expression, and promoting the generation and differentiation of TH2 cells. Additionally, neuropeptides can induce Treg cell production while suppressing autoreactive T cell activation through secretion of IL-10 and TGFβ. Secondly, neuropeptides directly influence macrophages by promoting their differentiation into the M2 phenotype. This enhances the expression of anti-inflammatory factors while suppressing pro-inflammatory factor expression. The red “X” means inhibit. The diagram of the immune cells was refined based on Krause’s work [18].

A previous study revealed that CD4^+^ and CD8^+^ TH2 immune cells are the primary sources of VIP in response to inflammatory stimuli or antigen activation [7]. Additionally, α-MSH is predominantly expressed in lymphocytes and monocytes, and its production is stimulated by inflammatory factors [19]. Research has shown that VIP and α-MSH act as potent anti-inflammatory agents, effectively inhibiting the production of inflammatory mediators (TNFα, IL-6, and IL-1β) and chemokines (CCL5, IL-8, and IP-10), downregulating inducible nitric oxide synthase (iNOS) expression, and thus diminishing nitric oxide (NO) release. Meanwhile, the activation of macrophages, microglia, and monocytes enhances the production of the anti-inflammatory cytokines IL-10 and TGFβ [7]. Concurrently, VIP and α-MSH play crucial roles in modulating the balance between TH1 cells and regulatory T cells within the organism (Figure 2). This regulation ensures a stable equilibrium between anti-inflammatory and pro-inflammatory factors, thereby reducing the risk of autoimmune diseases [7]. Moreover, neuropeptides can influence macrophages to effectively modulate the M1/M2 balance and enhance the body’s anti-inflammatory capacity (Figure 2) [20].

Additionally, UCN I, AM, and cortistatin, which are synthesized by nerve and immune cells (such as macrophages, monocytes, lymphocytes, or T cells), act as endogenous immunoregulatory factors with substantial anti-inflammatory properties [7]. They are capable of inhibiting the production of pro-inflammatory cytokines (TNFα, IL-6, IL-12, IL-1β, and MIF), chemokines (CCL5, IP-10, MIP-1α, MIP-2, MCP-1, and eotaxin), and NO, whereas they stimulate immune cells to produce the anti-inflammatory cytokine IL-10 [7].

### 2.2. Pro-Inflammatory Effects

The inflammatory response is a highly complex process involving numerous factors and signaling pathways. As a crucial component of the body’s defense and protective mechanisms, the inflammatory response is essential for eliminating harmful foreign substances and initiating self-repair processes [21]. Generally, regulated pro-inflammatory responses are not harmful; rather, they are crucial for maintaining homeostasis under normal conditions [22]. During the onset of an inflammatory response, certain neuropeptides act as immunomodulators by interacting with various receptors to facilitate the progression of inflammation [23]. Certain neuropeptides synthesized by neuronal and immune cells play a significant role in modulating pro-inflammatory responses; the primary neuropeptides involved include substance P (SP), calcitonin gene-related peptide (CGRP), and neuromedin U (NmU) [24,25].

**Figure 3:**
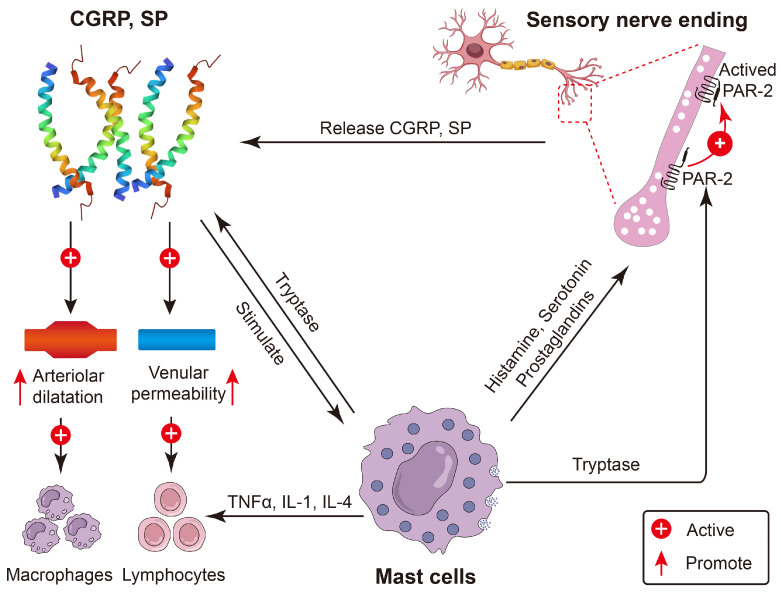
Antimicrobial neuropeptides are key players in pro-inflammation. Certain neuropeptides play a pivotal role in mediating neurogenic inflammation and augmenting inflammatory responses. In the presence of inflammatory stimuli, mast cells undergo degranulation, and through the action of neuropeptides, they elicit arterial dilation and heightened venous permeability, thereby establishing an environment conducive to inflammatory processes. Moreover, mast cell-derived tryptase possesses the ability to cleave activated PAR-2, thereby facilitating the release of neuropeptides and instigating the inflammatory response. The diagram of the immune cells was refined based on Krause’s work [18]. The diagram of the nerve cell was designed by pch.vector/Freepik.

Neuropeptides primarily trigger pro-inflammatory responses by stimulating or enhancing the expression of pro-inflammatory cytokines [26]. For example, SP and CGRP can promote the release of pro-inflammatory cytokines (TNFα, IL-1, and IL-4) and histamine from mast cells, thereby fostering an environment conducive to inflammation (Figure 3) [27,28,29,30]. Furthermore, neuropeptides can induce inflammation by regulating the balance between anti-inflammatory and pro-inflammatory factors. For example, SP downregulates the mTOR signaling pathway, reduces the expression of the anti-inflammatory cytokine IL-10, and enhances the release of the pro-inflammatory cytokines IL-12p40 and IL-23, which subsequently increases susceptibility and promotes inflammatory responses [31]. Recent research has indicated that NmU can be upregulated in response to pathogen infection, promoting type 2 cell responses and activating eosinophils, thus inducing pro-inflammatory effects [25].

## 3. Antimicrobial Neuropeptide Receptors as Potential Therapeutic Targets for Immune Diseases

Neuropeptides function as neuroendocrine regulators [6], with certain neuropeptides displaying antimicrobial properties that play crucial roles in immune regulation [7,23]. The neuropeptides under consideration include pituitary adenylate cyclase-activating polypeptide (PACAP), VIP, α-MSH, AM, neuropeptide Y (NPY), urocortin II (UCN II), CGRP, SP, and catestatin (CST). This study conducted a thorough analysis of the expression characteristics of these ten antimicrobial neuropeptides and their corresponding receptors (Table 1). Additionally, we performed a comprehensive systematic review of potential therapeutic agents targeting these receptors, along with an evaluation of their clinical viability in preclinical and/or clinical studies (Table 2).

### 3.1. Pituitary Adenylate Cyclase-Activating Polypeptide (PACAP)

PACAP is a neuropeptide consisting of 38 amino acids that was first isolated from the hypothalamus of sheep by Atsuro Miyata and colleagues in 1989 [109]. This neuropeptide is widely distributed throughout the nervous and immune systems, is predominantly found in thymic cells and lymphocytes, and is classified within the secretin/glucagon/VIP superfamily [32]. The receptors VPAC1, VPAC2, and PAC1 are utilized by both PACAP and VIP [110]. Although PACAP and VIP display similar affinities for VPAC1 and VPAC2 [111], the affinity of PACAP for PAC1 is approximately 300–1000 times greater than that of VIP [7]. VPAC1 is constitutively expressed in monocytes and T cells, whereas VPAC2 is predominantly present in thymocytes and mast cells. Conversely, PAC1 is expressed mainly in macrophages and dendritic cells (DCs) in the lung [33]. PACAP and VIP primarily exert immunoregulatory effects through their interaction with the VPAC1 or VPAC2 receptors, whereas the PAC1 receptor is involved mainly in the release of growth factors and neuroprotective mechanisms [111].

Research has indicated that PACAP has a significant anti-inflammatory effect. Specifically, PACAP suppresses pro-inflammatory TH1 and TH17 responses while simultaneously promoting TH2 and Treg responses [112]. Furthermore, studies on microglial inflammation have demonstrated that PACAP can inhibit the production of pro-inflammatory mediators, such as TNFα, IL-1β, IL-6, and NO, which are induced by LPS-activated microglia through its action on the VPAC1 receptor [113]. This action significantly reduces microglial activity. Studies have shown that PACAP and VIP inhibit the Janus kinase (JAK)/signal transducer and activator of the transcription 1 (STAT1)-signaling pathway, regulate *cd40* gene expression (a key mediator of inflammation), and reduce IFNγ-induced microglial inflammation [111]. In models of traumatic brain injury, PACAP has also been found to inhibit secondary inflammation in microglia and neurons through the TLR4 pathway, reducing neuronal death, easing inflammation, and facilitating the recovery of normal functions [114].

The antagonists of the VPAC1 receptor include PG 97-269 [58]. Noteworthy agonists are [R^16^]-PACAP (1-23), [L^22^]-VIP, and [Lys^15^, Arg^16^, Leu^27^]-VIP (1-7)-GRF (8-27)-NH2 [59,60]. Among these, PG 97-269 [58] has progressed to the pre-clinical research stage. PACAP enhances the sensitivity of the trigeminal nerve, which leads to vasodilation and subsequently initiates an inflammatory response. The VPAC1 antagonist PG 97-269 can partially block the vasodilation induced by PACAP [59]. Therefore, PG 97-269, as a VPAC1 target antagonist, could serve as a treatment for migraines triggered by the trigeminal nerve system [59]. However, its efficacy and safety profile require further validation through clinical trials [59]. Furthermore, research has indicated that PG 97-269 may reduce the production of cytokines (IL-1β and IL-6) by immune cells, such as monocytes, mast cells, and macrophages, whereas it also inhibits their chemotactic activity, thus holding therapeutic potential in the treatment of colitis [61].

The antagonists of the VPAC2 receptor include PG99-465 and VIpep-3 [62]. The agonists include LBT-3627, RO25-1553, RO25-1392, and BAY 55-9837 [62,63]. Among them, LBT-3627 [63] and RO25-1553 [115] have progressed to the pre-clinical research stage. Notably, LBT-3627 has been shown to increase Treg activity, promote the survival of dopaminergic neurons in the substantia nigra, and decrease microglial numbers, indicating its therapeutic potential for Parkinson’s disease (PD) [63]. RO25-1553 selectively binds to the VPAC2 receptor, which reduces the release of TNFα and IL-12 from macrophages and monocytes, thereby contributing to its role in immune regulation [115].

The PAC1 receptor antagonist includes PACAP 6-38 [64], whereas its agonist is Maxadilan [65]. Among them, PACAP 6-38 [64] and Maxadilan [65] have progressed to the pre-clinical research stage. In vitro studies have demonstrated that PACAP enhances the excitability of various neurons through the activation of extracellular signal-regulated kinase (ERK) [64]. PACAP 6-38 has the potential to ameliorate central sensitization in the trigeminal nucleus caudalis of rats suffering from chronic migraine, providing a promising avenue for treating this condition [64]. Maxadilan may offer antiatherosclerotic protection by functioning downstream of cholesterol-induced vascular inflammation, thus safeguarding against atherosclerosis in ApoE knockout (ApoE^−/−^) mice [65].

### 3.2. Vasoactive Intestinal Peptide (VIP)

VIP, which is composed of 28 amino acids, derives its name from its initial identification in the intestines and its role as a vasodilator [116]. VIP is a member of the secretin/glucagon/VIP superfamily, a group of antimicrobial neuropeptides synthesized by lymphocytes and nerve cells that exhibit extensive immune functions [117]. VIP is extensively distributed throughout the body, and as a neurotransmitter, it has been identified in various organs and tissues. Within the immune system, VIPs originate primarily from two sources: nerve terminals and immune cells [7]. A study identified CD4^+^ and CD8^+^ TH2 immune cells as the primary sources of VIP following inflammatory or antigenic stimulation [118]. The literature has established that VIP and PACAP interact with the same three receptors: VPAC1, VPAC2, and PAC1 [110].

Research indicates that VIP is a potent anti-inflammatory agent [7]. Similar to PACAP, its immune modulatory effects are mediated through the receptors VPAC1 and VPAC2 [111]. VIP inhibits activated macrophages from producing IL-12, induces CD86 expression on DCs and macrophages, and facilitates the aggregation of specific TH2 cells, thereby prolonging their survival and promoting a TH2-type immune response [7]. Additionally, research suggests that inhibiting VIP signal transduction may increase both the proliferation and functional activity of CD8^+^ T cells in the context of viral infections and lymphoma [119]. Furthermore, VIP effectively modulates the balance between TH2 and TH1 responses within the body, maintaining a stable equilibrium between anti-inflammatory and pro-inflammatory factors to prevent the onset of autoimmune diseases [7]. Antagonists and agonists targeting VIP-specific receptors are described in PACAP.

### 3.3. α-Melanocyte Stimulating Hormone (α-MSH)

Melanocortin peptides, which include α-MSH β-MSH, γ-MSH, and adrenocorticotropic hormone (ACTH), are produced by the hydrolysis of proopiomelanocortin (POMC) [120]. α-MSH is composed of 13 amino acids and is well known for its role in skin pigmentation [35]. The precursor gene *pomc* is expressed in lymphocytes, monocytes, DCs, and islet cells, and the production of α-MSH can be induced by inflammatory factors [34]. α-MSH predominantly exerts its effects via melanocortin receptors, which consist of five distinct subtypes: MC-1R, MC-2R, MC-3R, MC-4R, and MC-5R [35]. Research has shown that MC-1R, MC-3R, and MC-5R are involved in immune regulation and exhibit anti-inflammatory properties. While MC-1R and MC-3R are located primarily within the central nervous system, MC-5R is predominantly present in peripheral tissues and is extensively distributed among macrophages and lymphocytes [36].

Research confirms that α-MSH has anti-inflammatory properties [36]. Its role in immune regulation parallels that of VIP; however, α-MSH also stimulates the proliferation of CD4^+^ and CD25^+^ regulatory T cells, consequently promoting the production of another anti-inflammatory cytokine, TGFβ [7]. In vitro studies have demonstrated that during an inflammatory response, the expression of MC-1R is significantly increased, with α-MSH exerting a pronounced effect on the functionality of various cell types, including monocytes [121]. This effect includes the inhibition of NF-κB activation, which is a critical prerequisite for most inflammatory responses induced by inflammatory factors [121]. Additionally, MC-1R can exert anti-inflammatory effects by acting on the JAK–STAT pathway downstream, thereby activating the cAMP response element-binding protein (CREB), a transcription factor that binds to DNA and increases the expression of anti-inflammatory genes [122].

The antagonists of the MC-1R include Ac-DPhe(pI)-DArg-Nal (2′)-Arg-NH2 [66] and ASIP [67] among others. The agonists include PL8177 [68], MT-7117 [69], and BMS-470539 [70]. Among these, PL8177 [68] has progressed to the pre-clinical research stage and MT-7117 [69] has advanced to Phase 2 clinical trials. Notably, the agonist PL8177 has the potential to enhance macrophage efferocytosis but inhibits the release of pro-inflammatory cytokines such as IL-1β, IL-6, and TNFα through cAMP accumulation [68]. Consequently, it serves as a promising target for treating inflammation-related diseases, including arthritis [68]. MT-7117 also inhibits the activation of inflammatory cells, such as monocytes and macrophages, as well as inflammatory signals such as IL-6, thereby exerting anti-inflammatory effects [69]. Furthermore, MT-7117 has the capacity to suppress fibroblast activation, suggesting its potential as a therapeutic agent for systemic sclerosis. A Phase 2 clinical trial is currently in progress to evaluate the efficacy and tolerability of MT-7117 in patients with early-stage progressive diffuse cutaneous systemic sclerosis (ClinicalTrials.gov number, NCT04440592) [69].

For the MC-3R, the antagonist includes SHU-9119 [71], whereas agonists include g-2-MSH [72] and [D-Trp^8^]-γ-MSH [73], among others. The antagonist of the MC-5R receptor consists of x-Cha-DPhe-Arg-Trp-y and x-His-Nal (2′)-Arg-Trp-y [71]. Agonists for this receptor include SHU-9119 (Ac-Nle-c[Asp-His-D-Nal(2′)-Arg-Trp-Lys]-NH2) [71] and PG-901 [74]. Research indicates that selective activators of MC-5R could enhance therapeutic approaches for immune disorders; however, the precise mechanisms underlying this effect remain inadequately understood [74].

### 3.4. Ghrelin

Ghrelin is a neuropeptide composed of 28 amino acids that was first isolated from the gastric tissue of rats [123]. It is primarily secreted by human P/D1 cells and rat X/A-like cells [37]. Numerous studies have demonstrated that ghrelin regulates energy metabolism and the inflammatory response during the aging process by acting on its receptor, GHS-R [124]. The literature indicates that GHS-R, also known as GHS-R1a, is predominantly distributed in the thymus cells of both humans and mice. GHS-R1b, a truncated isoform of GHS-R1a lacking the sixth and seventh transmembrane domains, is coexpressed with GHS-R1a but does not bind to ghrelin. The in vivo expression of GHS-R1b primarily depends on its oligomerization with GHS-R1a [125]. GHS-R1b may have regulatory effects and can also facilitate the oligomerization of GHS-R1a with other receptors [126].

Ghrelin was identified in 1996, yet its role in immune regulation has remained insufficiently understood over the nearly 30 years since its discovery [127]. Although the majority of researchers consider ghrelin to be an anti-inflammatory neuropeptide [38], a subset of studies has confirmed that ghrelin can exert pro-inflammatory effects in the context of colitis [128]. Primarily, ghrelin demonstrates anti-inflammatory properties by suppressing the synthesis of pro-inflammatory cytokines, such as IL-6, IL-1β, and TNFα [38]. Notably, ghrelin has been shown to exert a significant anti-inflammatory effect on human endothelial cells, potentially through the inhibition of NF-κB activation [129]. Furthermore, in murine studies, ghrelin significantly downregulated the mRNA expression levels of the pro-inflammatory cytokines TNFα, IL-1β, and IL-6 in spinal microglia and infiltrating T cells [39]. However, research on colitis has shown that ghrelin can induce PKC-dependent NF-κB activation and IL-8 secretion in colon cells, subsequently promoting an inflammatory response [128].

There are several modulators of the GHS-R1a receptor, including antagonists such as [D-Lys^3^]-GHRP-6 [75] and L-756867 [76], as well as agonists such as MK-0677 [77], capromorelin [78], LY444711 [79], and GHRP-2 [80]. Inverse agonists include PF-05190457, AZ-GHS-22, and AZ-GHS-38 [81]. Among them, [D-Lys^3^]-GHRP-6 [75], L-756867 [76], LY444711 [79], GHRP-2 [80], and PF-05190457 [81] have progressed to the pre-clinical research stage, and capromorelin [78] has entered Phase 1 clinical trials. Among these compounds, the agonist LY444711 has been noted for its ability to inhibit the onset of brain inflammation and enhance cognitive function, positioning it as a potential therapeutic agent for Alzheimer’s disease [79]. Miriam Granado et al. demonstrated that the activation agent GHRP-2 significantly reduces IL-6 levels released by macrophages induced by LPS and inhibits inflammatory responses in rats with arthritis, suggesting that GHRP-2 may be a promising therapeutic candidate for targeting the GHS-R1a receptor for arthritis treatment [80]. In investigations of the inverse agonist of GHS-R1a, PF-05190457 displayed significant oral bioavailability and favorable tolerability, highlighting its considerable clinical potential for obesity treatment [81]. Moreover, the antagonist [D-Lys^3^]-GHRP-6 plays a role in regulating fasting glucose homeostasis [75], whereas L-756867 can suppress appetite, positioning both as potential pharmacological agents for weight management [76]. Capromorelin, a promising therapeutic compound for the management of spinal cord injury (SCI) constipation, has demonstrated an excellent safety profile in Phase 1 clinical trials [78].

### 3.5. Adrenomedullin (AM)

AM is a neuropeptide consisting of 52 amino acids and shares structural similarities with CGRP. It was first isolated from human pheochromocytoma in 1993 [130] and is classified within the amylin/intermedin/CGRP family [131]. Although AM was initially thought to function primarily as a vasodilator, recent studies have revealed its diverse physiological roles and remarkable anti-inflammatory properties. AM is expressed and secreted by DCs, and stimulating these cells to mature through lipopolysaccharide (LPS) exposure has been shown to increase AM expression [42]. Moreover, AMs can be synthesized by various cell types, including endothelial cells, fibroblasts, and epithelial cells. Its expression is upregulated in response to inflammatory conditions or hypoxia [41].

The AM receptor is a heterodimer composed of the calcitonin receptor-like receptor (CRLR) and receptor activity-modifying proteins (RAMPs), which are classified into three subtypes: RAMP1, RAMP2, and RAMP3. The binding affinity for CGRP is greater for the CRLR/RAMP1 complex, whereas the affinities for AM are greater for CRLR/RAMP2 (AM1) and CRLR/RAMP3 (AM2) [40]. The CRLR/RAMP complexes are predominantly localized in the brain, with CRLR/RAMP1 specifically identified in cerebral blood vessels and the trigeminal ganglion, among other regions [132]. Additionally, AM1 and AM2 are more extensively distributed throughout the brain and are primarily localized in the ventricles, trigeminal nerve, and choroid plexus [133].

AM acts as an endogenous immune regulator, demonstrating significant anti-inflammatory properties [7]. AM exerts these effects by inhibiting the synthesis of TNFα, IL-6, IL-12, IL-1β, and nitric oxide through macrophage activation [7]. Furthermore, AM can mediate its anti-inflammatory actions via the activation of NF-κB [134]. Research indicates that AM can alleviate inflammation in the early stages of pulmonary fibrosis by targeting the AM1 receptor and can also play a role in mediating fibroblast suppression during the progression of pulmonary fibrosis disease [135].

The antagonists of CRLR/RAMP1, AM1, and AM2 include olcegepant [82], telcagepant (MK-0974) [83], MK-3207 [84], and BMS-694153 [85]. AM22-52 and CGRP8-37 serve as potent antagonists of the AM1 receptor [97], whereas the antagonist SHF-638 demonstrates pronounced selectivity for the AM2 receptor [99]. Among them, telcagepant [83] has progressed to the pre-clinical research stage. Telcagepant, a potential therapeutic agent, targets CRLR/RAMP1 to inhibit the release of CGRP and alleviate pain [87]. Nevertheless, the observation of abnormal liver function resulting from its use compelled the cessation of clinical development [87]. Additionally, CRLR/RAMP1 antagonists include KB peptides (e.g., KBP-042, KBP-088, and KBP-089) [86], whereas the AM1 agonist is hAM1-52 [98] and the AM2 antagonist is AM2/IMD [86]. The study confirmed that the KB peptide effectively preserved the therapeutic potency of salmon CT, which is commonly used for treating Paget’s disease and osteoporosis in humans, while simultaneously enhancing its tolerability in mice [86].

### 3.6. Calcitonin-Gene Related Peptide (CGRP)

CGRP, which is composed of 37 amino acids, is a member of the calcitonin family [136]. It functions as an immune regulator and is widely distributed throughout the nervous system [137] and the immune system. CGRP plays a crucial role in modulating immune responses in macrophages [138], DCs, T cells, and various other immune cell types [43]. CGRP is involved in a range of physiological functions within the body, including vasodilation [139], and exerts its immunoregulatory effects through interactions with specific receptors. Both CGRP and AM belong to the calcitonin family, which shares similarities in their receptors, which form a heterodimer consisting of CRLR and PAMRs (PAMP1, PAMP2, and PAMP3). Notably, the CRLR/PAMP1 complex has a relatively high affinity for CGRP [40].

The regulatory role of CGRP in inflammatory responses remains a subject of debate, with some researchers contending that it promotes pro-inflammatory effects, whereas others assert anti-inflammatory effects. Some studies indicate that CGRP can facilitate the onset of inflammatory responses. For example, in macrophages infected with HSV-1,CGRP either alone or in combination with SP, the secretion of the pro-inflammatory cytokines IL-1β and TNF was significantly increased [47]. CGRP has the capacity to activate mast cells, promoting the release of histamine, which in turn increases vascular permeability and fosters an environment conducive to inflammation (Figure 3) [27]. Research on satellite glial cells has shown that CGRP can induce the expression of pro-inflammatory genes and facilitate the release of IL-1β. However, the molecular mechanisms through which CGRP mediates its pro-inflammatory effects on satellite glial cells remain inadequately understood [140].

Studies have indicated that CGRP functions as an anti-inflammatory neuropeptide [141]. In DCs, CGRP inhibits the secretion of pro-inflammatory cytokines, including TNFα and IL-12 [44], thereby suppressing IL-12 production and hindering TH1 cell differentiation [142]. Furthermore, when human bronchial epithelial cells (HBECs) are pretreated with ovalbumin, T cell proliferation displays a dose-dependent association. Here, CGRP not only inhibits T cell proliferation but also reduces the secretion of the pro-inflammatory cytokine IFNγ [143], whereas it modulates IL-17 expression to enhance Th17-mediated inflammatory responses [144]. Additionally, CGRP has been found to suppress the release of pro-inflammatory mediators such as IL-8, the chemokine CCL2, and the chemokine CXCL1 in human endothelial cells. Simultaneously, it promotes the secretion of the anti-inflammatory cytokine IL-10 by macrophages and trigeminal glial cells [45]. In experiments conducted on a mouse model of colitis, the knockout of CGRP or the administration of CGRP antagonists resulted in increased susceptibility to colitis in the mice, thereby indirectly reinforcing the anti-inflammatory properties of CGRP [141]. Antagonists and agonists targeting CGRP-specific receptors are described in AM.

### 3.7. Substance P (SP)

SP is a peptide composed of 11 amino acids and was first isolated from the brains and intestines of equines by Von Euler and Gaddum in 1931 [145]. As an immunomodulator, SP is primarily secreted by neurons and produced by specific inflammatory cells, including macrophages and DCs [146]. Research has shown that SP is encoded by the *tac1* gene and is expressed in various cell types, such as microglia, macrophages, DCs, and epithelial cells [33]. SP belongs to the tachykinin family, which includes three primary receptors: NK1R, NK2R, and NK3R. Among these, NK1R is widely distributed throughout both the central and peripheral nervous systems, whereas NK2R and NK3R are mainly localized within the peripheral nervous system [33].

Research indicates that SP is a pro-inflammatory neuropeptide that plays a critical role in immune regulation through its specific binding to the neurokinin-1 receptor (NK1R) on cells [146]. In activated human T lymphocytes, SP enhances the expression of the chemokine MIP-1β via an NF-κB-mediated pathway, facilitating the migration of T lymphocytes to sites of inflammation [33]. In mast cells, SP promotes the secretion of pro-inflammatory mediators, including TNFα, IL-1, and IL-4, which subsequently affect macrophages and lymphocytes [28,147] (Figure 3). Additionally, SP stimulates endothelial cells to release NO, thereby initiating inflammatory responses [148]. Moreover, SP has been shown to increase the expression of the pro-inflammatory cytokines IL-12p40 and IL-23 while concurrently suppressing the production of the anti-inflammatory cytokine IL-10. In this manner, it plays a pivotal role in immune regulation by facilitating inflammatory responses [33].

Antagonists of NK1R include aprepitant [103], L-733060 [104], lanepitant [105], and befetupitant [105], whereas the agonist GR73632 [106]. Among them, aprepitant [103] has progressed to the clinical research stage. Aprepitant is efficacious in the management of chemotherapy-induced nausea and vomiting (CINV) and represents a promising therapeutic strategy when combined with various targeted anticancer agents [149]. To date, Aprepitant has been approved by the U.S. Food and Drug Administration (FDA) for low-dose administration to manage chemotherapy-induced nausea and vomiting [150]. Furthermore, aprepitant has been shown to facilitate the polarization of M2 microglia via the PKC/p38MAPK/NF-κB-signaling pathway and mitigate hemorrhagic areas, and has emerged as a promising therapeutic agent for treating posthemorrhagic brain conditions [103].

### 3.8. Neuropeptide Y (NPY)

NPY, composed of 36 amino acid residues, was first isolated from porcine brain tissue in 1982 and is classified within the neuroendocrine peptide (NPY) family [151]. NPY is recognized as the most abundant neuropeptide in both the central and peripheral nervous systems [152], exhibiting widespread distribution throughout the body, including the intestines, thymus, smooth muscle, and various other tissues [153]. Notably, it can be synthesized and secreted by immune cells such as monocytes, lymphocytes, and NK cells [152]. NPY interacts with six receptors: Y1R, Y2R, Y3R (yet to be cloned in mammals), Y4R, Y5R, and Y6R [51]. The primary receptors expressed in the human body include Y1R, Y2R, Y4R, and Y5R [154]. Notably, Y1R is found across nearly all immune cell types, whereas the distributions of Y2R, Y4R, and Y5R are predominantly observed in neutrophils. Current research highlights the significant roles of Y1R and Y2R in immune regulation [51].

NPY serves as a crucial immune regulator, exerting anti-inflammatory effects through its interaction with Y1R [152]. Most studies indicate that NPY plays a pivotal role in immune regulation via two primary mechanisms. First, NPY acts as an immune mediator, influencing immune cells through autocrine or paracrine mechanisms [152]. For example, the presence of Y1R antagonists or a deficiency in Y1R results in the reduced secretion of IL-12 and TNFα by activated macrophages, suggesting that NPY released from these macrophages is essential for the production of pro-inflammatory factors [155]. Furthermore, NPY has direct effects on immune cells via its receptors [51]. Research indicates that NPY enhances the secretion of IL-1β in human peripheral blood monocytes and mouse macrophages while simultaneously inhibiting the release of the pro-inflammatory cytokine TNFα from LPS-stimulated macrophages. Additionally, it promotes the production of the anti-inflammatory factor TGF-β1, thereby clarifying its role in mitigating inflammatory responses [152]. In microglia, NPY can reduce the secretion of the pro-inflammatory cytokines IL-1β and TNFα through the activation of Y1R [51]. Moreover, findings from animal studies indicate that NPY (10^−9^ M) effectively stimulates immature DCs to upregulate the expression of IL-6 and IL-10 via Y1R, enhances the secretion of IL-4 via TH2 polarization, and suppresses IFNγ secretion by these cells [156].

Y1R antagonists include BIBP3226 [88], BIB03304 [89], and 1229U91 [90]. Agonists include [Leu^31^,Pro^34^]NPY [89] and [Pro^30^,Nle^31^,Bpa^32^,Leu^34^]NPY (28-36) [91]. Among them, [Leu^31^,Pro^34^]NPY [89] and IBP3226 [88] have progressed to the pre-clinical research stage. The agonist [Leu^31^, Pro^34^]NPY has been shown to inhibit gastric acid secretion, reduce gastric mucosal injury, impede the progression of gastric lesions, and may serve as a potential therapeutic agent for gastric disorders [89]. NPY interacts with Y1R to suppress the release of IL-1β and TNFα from microglia; the antagonist BIBP3226 can decrease the immune activity of microglia by inhibiting NPY through Y1R [88].

Y2R antagonists include SF-11 [92], JNJ-31020028 [93], and BIIE0246 [94]. Agonists include PYY(3-36) [89,95], NPY(13-36) [89], and obinepitide (TM30338) [96]. Among them, PYY(3-36) [89,95] has progressed to the pre-clinical studies stage. In a murine model of acute pancreatitis, the peptide PYY(3-36) has been demonstrated to increase pancreatic cell proliferation while concurrently inhibiting amylase secretion (a biomarker for pancreatitis) [89]. In murine models of colitis, treatment with the PYY(3-36) agonist significantly reduced the levels of myeloperoxidase (MPO), TNFα, and IL-6 in affected mice [95]. Therefore, PYY(3–36) can be considered a promising candidate for the pharmacological management of both acute pancreatitis and colitis.

### 3.9. Urocortin II (UCN II)

UCN II is a peptide consisting of 38 amino acids [100] and is distributed throughout the brain, small intestine, heart, and skeletal muscle [157]. It can be detected in immune cells, including macrophages, endothelial cells, and fibroblasts, in humans [54]. UCN II is classified as a member of the corticotropin-releasing factor (CRF) family, which also includes UCN I. This family consists of two receptors: CRFR1 and CRFR2 [100]. CRFR1 is primarily localized in the brainstem and hypothalamus, whereas CRFR2 shows a more restricted distribution within the central nervous system and is also expressed in peripheral tissues, such as the heart and skeletal muscle [158]. Research indicates that UCN I has comparable affinity for both CRFR1 and CRFR2, whereas UCN II has a binding preference for CRFR2 and exhibits negligible affinity toward CRFR1 [100].

Numerous experimental studies have demonstrated that UCN II functions as an anti-inflammatory neuropeptide. In a study on intestinal inflammation, the knockout of CRFR2 resulted in exacerbated intestinal inflammation and increased mortality rates in experimental mice. UCN II produced by CD141^+^ DCs (dendritic cast-off cells) was found to facilitate the generation of functionally inhibitory Tregs and effectively suppress allogeneic T cell-mediated skin inflammation in a murine model [54]. UCN II acts by specifically targeting CRFR2, leading to the rapid induction of apoptosis in macrophages. This process inhibits the release of TNFα but enhances the production of IL-10, ultimately contributing to its anti-inflammatory effects [100]. Furthermore, UCN II may also mediate anti-inflammatory effects by increasing the expression and activation of STAT3 while simultaneously suppressing the expression and activation of STAT1 [159].

CRFR2 antagonists, such as anti-sauvagine30 [100] and astressin 2B [101], and agonists such as CT38s [102], have distinct effects. Anti-sauvagine30, a specific antagonist, effectively blocks the pro-apoptotic effects of CRFR2 [100]. Among these, astressin 2B [101] has progressed to the pre-clinical research stage, and CT38s [102] has entered clinical trials. Astressin 2B can downregulate inflammatory mediators such as keratinocyte chemokines and monocyte chemoattractant protein 1, helping to prevent inflammation in the intestinal tract [101]. The agonist CT38s induces dose-dependent changes in norepinephrine and corticosterone release, along with effects on gastrointestinal motility, urine output, heart rate (HR), and mean arterial pressure (MAP), and the clinical trials are underway (ClinicalTrials.gov number, NCT03613129) [102].

### 3.10. Catestatin (CST)

CST is a 21-amino acid peptide derived from chromogranin A (CgA) that acts on adrenal chromaffin cells and effectively inhibits catecholamine secretion [160]. Studies have shown that CST has various physiological functions, including vasodilation and immune regulation [161]. It primarily interacts with the type 2 muscarinic acetylcholine receptor (M2 receptor) [54] and nicotinic acetylcholine receptor (nAChR) [162]. AChRs are therapeutic targets for several neurological disorders, and the M2 receptor is widely distributed in brain tissues [163].

Research suggests that CST functions as an anti-inflammatory neuropeptide. Feng et al. reported that CST activates the M2 receptor, triggering the ERK1/2 and PI3K/Akt pathways, which reduce stress-induced cell apoptosis and inflammation-related tissue damage [55]. CST also limits immune cell infiltration in inflamed tissues and promotes the differentiation of macrophages into anti-inflammatory phenotypes [56]. Additionally, CST inhibits the expression of pro-inflammatory factors such as TNFα and IL-1β and modulates M1 macrophage markers such as Mcp1 and iNOS to exert anti-inflammatory effects [57].

M2 receptor antagonists include trospium chloride, which is used to treat overactive bladder [107]. Trospium chloride [107] has entered clinical trials (ClinicalTrials.gov number, NCT03697252). Further large-scale and extended-duration trials are necessary to establish the safety and efficacy of this drug in patients with schizophrenia [107]. Agonists such as iperoxo [108] and xanomeline [107] hold the potential for treating conditions such as Alzheimer’s disease [107].

## 4. Future Perspectives and Conclusions

The nervous system is closely associated with the immune system and helps maintain immunological balance in both health and disease [164]. Investigating the immunoregulatory roles of neuropeptides, a key biochemical language shared by both systems has become increasingly important, especially in relation to therapies targeting their receptors in preclinical and/or clinical studies. Existing evidence indicates that neuropeptides likely play a crucial role in modulating immune responses and neuroinflammatory processes [165,166].

In this review, we mainly examined 10 antimicrobial neuropeptides, summarizing their expression characteristics and the immunomodulatory mechanisms mediated by their specific receptors. Importantly, the majority of these antimicrobial neuropeptides (eight out of ten) also exhibit significant anti-inflammatory properties, thereby contributing to the maintenance of tolerance in various immune disorders. Consequently, the combined antimicrobial and anti-inflammatory activities of neuropeptides could play a pivotal role in fortifying the defense mechanisms of the elimination of pathogens by the central nervous system (CNS) and the preservation of immune homeostasis. Moreover, these antimicrobial neuropeptides play a pivotal role in the pathogenesis and progression of neurodegenerative and metabolism-associated diseases. However, although these antimicrobial neuropeptides have exhibited significant clinical therapeutic potential, the majority of studies have been performed in animal models [12]. Therefore, caution is advised when extrapolating these findings to human diseases. Additionally, we explored potential drugs that target these antimicrobial neuropeptide receptors. However, the preferred strategy of pharmaceutical companies is to develop metabolically stable analogs, which is crucial for successful clinical application. In this case, we recommend exploring modifications to these analogs (such as single amino acid substitutions) or encapsulating these analogs within micelles or nanoparticles [12] to enhance metabolic stability and reduce degradation rates.

## Figures and Tables

**Table 1:** Antimicrobial neuropeptides: expression, receptors, and function in the body.

Name	Amino Acid Sequences	Immune Source	Immune Function	Receptor: Immune Cell	Receptor Type	Receptor Expression	Refs.
PACAP/VIP	HSDGIFTDSYSRYRKQMAVKKYLAAVLGKRYKQRVKNK-NH2	PACAP: Thymus cells, Lymphocytes, Plasma cells; VIP: Mast cells, Granulocytes, Lymphocytes	Inhibit IL-6, TNFα, NO; promote IL-10	Lymphocytes, monocytes, mast cells	VPAC1 VPAC2 PAC1	VPAC1: monocytes, T cells VPAC2: thymic cells, mast cells PAC1: macrophages and lung dendritic cells	[32,33]
α-MSH	HADGVFTSDFSKLLGQLSAKKYLESLM-NH2	Lymphocytes, Monocytes, Dendritic cells, and Islet cells	Inhibit IL-1, IL-6, TNFα, IL-2, IFNγ, IL-4, IL-13; promote IL-10	Lymphocytes, monocytes, dendritic cells, endothelial cells	MC-1R MC-2R MC-3R MC-4R MC-5R	MC-1R: monocytes, macrophages, dendritic cells (DCs), fibroblasts, inflammatory cells; MC-3R: central nervous system; MC-5R: macrophages, lymphocytes;	[34,35,36]
Ghrelin	GSSFLSPEHQRVQQRKESKKPPAKLQPR	Human P/D1 cells in rat X/A-like	Inhibit TNFα, IL-1β, IL-6	T lymphocytes, B lymphocytes, and neutrophils	GHS-R1a GHS-R1b	GHS-R1a: mononuclear cells; GHS-R1b: mononuclear cells	[37,38,39]
AM	YRQSMNNFQGLRSFGCRFGTCTVQKLAHQIYQFTDKDKDNVAPRSKISPQGY-NH2	Macrophages, Monocytes, and T cells; Lymphatic organs and Gastrointestinal tract	Inhibit TNFα, IL-6, IL-12, IL-1β, and NO	Macrophages	CRLR/RAMP2(AM1), CRLR/RAMP3(AM2)	AM1: fibroblasts	[40,41,42]
CGRP	ACDTATCVTHRLAGLLSRSGGVVKNNFVPTNVGSKAF-NH2	Nerve cell	Inhibit TNFα, IL-12, IFNγ; promote IL-4, IL-8, IL-10	Macrophages, dendritic cells (DCs), T cells, etc.	CRLR/RAMP1	Macrophages, dendritic cells (DCs), T cells, etc.	[43,44,45]
SP	RPKPQQFFGLM-NH2	Nerve cell, Inflammatory cells (e.g., Macrophages, Dendritic cells, etc.)	Inhibit IL-10; promote IL-1β, TNF, MIP-1β, IL-6	Microglia, macrophages, dendritic cells, etc.	NK1R	Microglia, macrophages, dendritic cells, etc.	[46,47,48]
NPY	YPSKPDNPGEDAPAEDMARYYSALRHYINLITRQRY-NH2	Activated Macrophages and Epithelial cells	Inhibit microglial cell TNFα, IL-1β; inhibit DCs IFNγ; promote human monocyte IL-1β	Monocytes, lymphocytes, granulocytes, etc.	Y1R Y2R Y3R Y4R Y5R Y6R	Y1: All types of immune cells; Y2R, Y4R, Y5R: neutrophile granulocytes; Y6R: mice	[49,50,51,52]
UCN II	VILSLDVPIGLLRILLEQARYKAARNQAATNAQILAHV-NH2 (mouse)	Nerve cell	Inhibit TNFα; promote IL-10	Immune cells (such as macrophages), endothelial cells, and fibroblasts	CRH-R2	CRH-R2: macrophages	[53,54]
CST	SSMKLSFRARAYGFRGPGPQL	Nerve cell, Immune cell, Neuroendocrine cell	Inhibit TNFα, IL-1β; promote IL-4, IL-10	Mononuclear cells, macrophages, etc.	Type 2 muscarinic acetylcholine receptor	Mononuclear cells, macrophages, etc.	[55,56,57]

**Table 2:** The potential therapeutic agent for the receptor and therapeutic potential in preclinical and/or clinical studies.

Selective Receptor	Specific Ligand	Antagonist	Agonist	Disease
VPAC1	PACAP, VIP	PG 97-269 ([Acetyl-His^1^,D-Phe^2^,Lys^15^,Arg^16^,Leu^17^]VIP(3-7)/GRF(8-27)) [58]	[R^16^]-PACAP(1-23)[L^22^]-VIP, [Lys^15^,Arg^16^,Leu^27^]-VIP(1-7)-GRF(8-27)-NH2 [59,60]	Migraine [59] Colitis [61]
VPAC2	PACAP, VIP	PG99-465 [62], VIpep-3 [62]	LBT-3627 [63], RO25-1553 [62], RO25-1392 [62], BAY55-9837 [62]	PD (Parkinson’s disease) [63]
PAC1	VIP	PACAP 6-38 [64]	Maxadilan [65]	
MC-1R	α-MSH	[Ac-DPhe(pI)-DArg-Nal(2′)-Arg-NH2] [66], ASIP [67]	PL8177 (Ac-Nle^1^-cyclo (Glu^2^-L-His^3^-D-Phe^4^-Arg^5^-Dap^6^)-Trp^7^-NH_2_) [68], MT-7117 [69], BMS-470539 [70]	Arthrophlogosis [68]; Systemic sclerosis [69]
MC-3R	α-MSH	SHU-9119 (Ac-Nle-c[Asp-His-D-Nal(2′)-Arg-Trp-Lys]-NH2) [71],	g-2-MSH [72], [D-Trp8]-γ-MSH [73]	
MC-5R	α-MSH	x-Cha-DPhe-Arg-Trp-y, x-His-Nal(2′)-Arg-Trp-y [70]	SHU-9119 [71], PG-901 (Ac-Nle^4^-c[Asp^5^-Pro^6^-*D*Nal(2′)^7^-Arg^8^-Trp^9^-Lys^10^]-NH_2_) [74]	
GHS-R1a	Ghrelin	[D-Lys^3^]-GHRP-6 [75], L-756867 (H_2_N,D-Arg,Pro,Lys,Pro,D-Phe,Gln,D-Trp,Phe,D-Trp,Leu, Leu,NH_2_) [76]	MK-0677 [77], Capromorelin [78], LY444711 [79], GHRP-2 [80]	Alzheimer’s Disease [79]; Arthrophlogosis [80]; fat [81]
CRLR/RAMP1	CGRP	Olcegepant [82], Telcagepant (MK-0974) ((3*R*,6*S*)-3-Amino-6-(2,3-difluorophenyl) azepan-2-one) [83], MK-3207 (2-[(8*R*)-8-(3,5-difluorophenyl)-10-oxo-6,9-diazaspiro [4.5]dec-9-yl]-*N*-[(2*R*)-2′-oxo-1,1′,2′,3-tetrahydrospiro[indene-2,3′-pyrrolo [2,3-*b*]pyridin]-5-yl]acetamide) [84], BMS-694153 ((*R*)-4-(8-Fluoro-2-oxo-1, 2-dihydroquinazolin-3(4 H)-yl)-*N*-(3-(7-methyl-1*H*-indazol-5-yl)-1-oxo-1-(4-(piperidin-1-yl)piperidin-1-yl)propan-2-yl)piperidine-1-carboxamide) [85]	KBP-042, KBP-088, KBP-089 [86]	Migraine [87]
Y1R	NPY	BIBP3226 (*N*-[(1*R*)]-4-[(Aminoiminomethyl)amino-1-[[[(4-hydroxyphenyl)methyl]amino]carbonyl]butyl-α-henylbenzeneacetamide trifluoroacetate) [88], BIB03304 [89], 1229U91 [90]	[Leu^31^,Pro^34^]NPY [89], [Pro^30^,Nle^31^,Bpa^32^,Leu^34^]NPY(28-36) [91]	Gastric diseases [89]
Y2R	NPY	SF-11 ([*N*-(4-ethoxyphenyl)-4-(hydroxydiphenylmethyl)-1-piperidinecarbothioamide]) [92], JNJ-31020028 [93], BIIE0246 ((*S*)-*N*2-[[1-[2-[4-[(*R*,*S*)-5,11-dihydro-6(6*h*)-oxodibenz[*b*,*e*]azepin-11-yl]-1-piperazinyl]-2-oxoethyl]cyclopentyl]acetyl]-*N*-[2-[1,2-dihydro-3,5(4*H*)-dioxo-1,2-diphenyl-3*H*-1,2,4-triazol-4-yl]ethyl]-argininamide) [94]	PYY(3-36) [89,95], NPY(13-36) [89], Obinepitide (TM30338) [96]	Acute pancreatitis and colitis [89,95]
AM1	AM	Olcegepant [82], Telcagepant (MK-0974) [83], MK-3207 [84], BMS-694153 [85], AM22-52 [97], CGRP8-37 [97]	hAM1-52 [98]	Migraine [87]
AM2	AM	Olcegepant [82], Telcagepant (MK-0974) [83], MK-3207 [84], BMS-694153 [85], SHF-638 [99]	AM2/IMD [86]	Migraine [87]
CRFR2	UCN II	Anti-sauvagine30 [100], Astressin 2B [101]	CT38s [102]	Intestinal inflammation [101]
NK1R	SP	Aprepitant [103], L-733060 [104], Lanepitant [105], Befetupitant [105]	GR73632 (δAva[l-Pro^9^, *N*-MeLeu^10^]SP-(7-11)), septide ([pGlu^6^,Pro^9^]SP-(6-11)) [106]	Cerebral hemorrhage [103]
M2	CST	Trospium chloride [107]	Iperoxo [108], Xanomeline [107]	Alzheimer’s Disease [107]; overactive bladder [107]

## Data Availability

Not applicable.

## Abbreviations

The following abbreviations are used in this manuscript:

α-MSH	α-melanocyte-stimulating hormone
ACTH	Adrenocorticotropic hormone
AM	Adrenomedullin
AMPs	Antimicrobial peptides
ApoE^−/−^	ApoE knockout
CgA	Chromogranin A
CGRP	Calcitonin gene-related peptide
CINV	Chemotherapy-induced nausea and vomiting
CNS	Central nervous system
CREB	cAMP response element-binding protein
CRF	Corticotropin-releasing factor
CRLR	Calcitonin receptor-like receptor
CST	Catestatin
DCs	Dendritic cast-off cells
DCs	Dendritic cells
ERK	Extracellular signal-regulated kinase
FDA	Food and Drug Administration
HANS	Hypothalamic-autonomic nervous system axis
HBECs	Human bronchial epithelial cells
iNOS	Inducible nitric oxide synthase
JAK	Janus kinase
LPS	Lipopolysaccharide
MAP	Mean arterial pressure
MK-0974	Telcagepant
MPO	Myeloperoxidase
NAChR	Nicotinic acetylcholine receptor
NmU	Neuromedin
NO	Nitric oxide
NPY	Neuropeptide Y
PACAP	Pituitary adenylate cyclase-activating polypeptide
PD	Parkinson’s disease
POMC	Proopiomelanocortin
RAMPs	Receptor activity-modifying proteins
SCI	Spinal cord injury
SP	Substance P
STAT1	Signal transducer and activator of transcription 1
TM30338	Obinepitide
UCN II	Urocortin II
VIP	Vasoactive intestinal peptide
